# Regulation of *ABI5* expression by ABF3 during salt stress responses in *Arabidopsis thaliana*

**DOI:** 10.1186/s40529-019-0264-z

**Published:** 2019-08-09

**Authors:** Hui-Chun Chang, Min-Chieh Tsai, Sih-Sian Wu, Ing-Feng Chang

**Affiliations:** 1 Institute of Plant Biology, National Taiwan University, Taipei, Taiwan; 2 Department of Life Science, National Taiwan University, Taipei, Taiwan; 3 Genome and Systems Biology Degree Program, National Taiwan University and Academia Sinica, Taipei, Taiwan

**Keywords:** ABF3, Calcium, 14-3-3, ABI5, Salt, Abscisic acid, Phosphorylation

## Abstract

**Background:**

Basic region/leucine zippers (bZIPs) are transcription factors (TFs) encoded by a large gene family in plants. ABF3 and ABI5 are Group A bZIP TFs that are known to be important in abscisic acid (ABA) signaling. However, questions of whether ABF3 regulates *ABI5* are still present.

**Results:**

In vitro kinase assay results showed that Thr-128, Ser-134, and Thr-451 of ABF3 are calcium-dependent protein kinase phosphorylation sites. Bimolecular fluorescence complementation (BiFC) analysis results showed a physical interaction between ABF3 and 14-3-3ω. A Thr-451 to Ala point mutation abolished the interaction but did not change the subcellular localization. In addition, the Arabidopsis protoplast transactivation assay using a luciferase reporter exhibited *ABI5* activation by either ABF3 alone or by co-expression of ABF3 and 14-3-3ω. Moreover, chromatin immunoprecipitation-qPCR results showed that in Arabidopsis*, ABI5* ABA-responsive element binding proteins (ABREs) of the promoter region (between − 1376 and − 455) were enriched by ABF3 binding under normal and 150 mM NaCl salt stress conditions.

**Conclusion:**

Taken together, our results demonstrated that *ABI5* expression is regulated by ABF3, which could contribute to salt stress tolerance in *Arabidopsis thaliana*.

**Electronic supplementary material:**

The online version of this article (10.1186/s40529-019-0264-z) contains supplementary material, which is available to authorized users.

## Background

Salt stress has recently become a serious problem that causes decreased crop yields worldwide and is caused by global climate change. The components of salt stress signaling in plants have, therefore, become important topics in recent years. The Salt Overly Sensitive (SOS) pathway was activated to confer salt tolerance in plants under conditions of salt stress (Zhu 2001; Munns and Tester 2008). In addition, many genes have been shown to be transcriptionally up-regulated under salt or osmotic stress conditions (Shinozaki and Yamaguchi-Shinozaki 2007). These signal transduction pathways included the abscisic acid (ABA)-independent (Shinozaki and Yamaguchi-Shinozaki 2007) and ABA-dependent pathways (Shinozaki and Yamaguchi-Shinozaki 2007; Fujita et al. 2011). In the ABA-dependent pathway, the ABA receptor serves the first line of ABA signal perception (Kline et al. 2010). This pathway induces the bZIP transcription factor (TF), which binds to the ABA-responsive element (ABRE) for the up-regulation of downstream genes, such as *RD29B* (Shinozaki and Yamaguchi-Shinozaki 2007). By contrast, the ABA-independent pathway induces expression of a transcription factor gene, *DREB2*. DREB2 belongs to the DRE BINDING PROTEIN/C-REPEAT BINDING FACTOR family that binds to the dehydration-responsive element/C-repeat (DRE/CRT) element. *RD29A* gene activation is both ABA-dependent and ABA-independent. DREB transcription factor binds to the DRE/CRT element of a downstream gene, such as the *RD29A* gene, which results in up-regulation of this gene under drought stress, which eventually leads to enhanced salt or osmotic stress tolerance in plants.

bZIP transcription factors are found in animals, yeast, and plants. In Arabidopsis, there are 75 bZIP members in the bZIP family (Jakoby et al. 2002). The bZIP family can be divided into ten groups (A, B, C, D, E, F, G, H, I, and S). In maize, 125 bZIP genes encode 170 bZIP proteins, and based on phylogenetic analysis results; these can be divided into 11 groups (Wei et al. 2012). Based on the primary structure, each bZIP TF has a basic region for DNA binding, and a leucine zipper domain (Jakoby et al. 2002). bZIP TFs are G-box binding factors (GBFs), which can bind the G-box motif on DNA with ACGT *cis*-elements (Foster et al. 1994; Sibéril et al. 2001). The ABRE motif belongs to the G-box family (Fujita et al. 2011). bZIP TFs can dimerize to form homodimers and heterodimers (Deppmann et al. 2004; Vinson et al. 2006), and the homodimers can be visualized using bimolecular fluorescence complementation (BiFC) (Walter et al. 2004). bZIP TFs can also interact with the 14-3-3 scaffold signaling protein to provide signals (Sibéril et al. 2001; Eckardt et al. 2001; Schoonheim et al. 2007; de Boer et al. 2013; Vysotskii et al. 2013).

*bZIP* genes have been reported to be involved in the abiotic stress response (Uno et al. 2000). bZIP transcription factors can be membrane-bound and released into the cytosol during stress responses (Seo et al. 2008). In Arabidopsis, *ABF3* and *ABF4* are involved in ABA signaling (Kang et al. 2002) and the salt stress response (Kim et al. 2004). Activated AtbZIP17 was shown to enhance salt tolerance (Liu et al. 2008). In rice (*Oryza sativa*), constitutive *OsbZIP46* activation conferred drought stress tolerance (Tang et al. 2012). Overexpression of the soybean (*Glycine max*) *GmbZIP1* gene improved high salt stress tolerance in transgenic plants (Gao et al. 2011), and a maize (*Zea mays*) *ZmbZIP72* gene conferred salt stress tolerance in Arabidopsis transgenic plants (Ying et al. 2012). Overexpression of the rice *OsbZIP23* gene and the tomato (*Solanum lycopersicum*) *SIAREB* gene improved drought and high salt stress tolerance in the respective transgenic plants (Xiang et al. 2008; Hsieh et al. 2012). In lotus (*Nelumbo nucifera*) plants, the *LrbZIP* gene was shown to be important in the salt resistance of roots (Cheng et al. 2012). However, the molecular mechanisms of the *bZIP* genes in salt stress responses is still not completely understood.

The abscisic acid responsive element-binding factor 3 (ABF3) is a member of the group A bZIP TFs. *ABF3* overexpression in Arabidopsis showed an ABA hypersensitive phenotype (Kang et al. 2002) and increased drought stress tolerance in both rice and alfalfa (Oh et al. 2005; Wang et al. 2016). Ectopic Arabidopsis *ABF3* expression conferred drought tolerance in soybeans (Kim et al. 2018) and cotton (*Gossypium hirsutum*) (Kerr et al. 2018). However, the target genes of *ABF3* have not been thoroughly studied. Therefore, in this study, we investigated the transcriptional regulation of *ABI5,* another group A bZIP member, by the ABF3 and 14-3-3 proteins in Arabidopsis. The bimolecular fluorescence complementation (BiFC) assay was used to confirm the interaction between ABF3 and 14-3-3ω, and the transactivation assay was used to investigate if ABF3 regulates *ABI5*. Promoter deletion was also tested in the transactivation assay. Moreover, chromatin immunoprecipitation-qPCR was also introduced. Our results showed regulation of *ABI5* expression by ABF3 in response to salt stress in *Arabidopsis thaliana.*

## Materials and methods

### Plant materials, growth conditions, and salt stress treatment

*Arabidopsis thaliana* ecotype *Columbia* was used in the present study. A T-DNA insertion mutant line, *abi5,* was obtained from Dr. Hsu-Liang Hsieh‘s lab at the National Taiwan University. A T-DNA insertion mutant line, *abf3* (SALK_096965) of *ABF3* gene (At4g34000), was ordered from the Arabidopsis Biological Resource Center (ABRC) (Additional file 1). Plant transformation was performed in Arabidopsis Col-0 using the floral dip method (Clough and Bent 1998). To perform the chromatin immunoprecipitation (ChIP) analysis, *AtABF3* overexpression lines were generated. Full-length *AtABF3* CDS driven by the 35S promoter in pEarleyGate103 vector was transformed into the Col-0 WT. *AtABF3* overexpression lines were isolated using the plant selection maker, BASTA. Seeds were surface sterilized and stratified at 4 °C for 3 days in the dark, then propagated and grown on 1/2 Murashige–Skoog (MS) agar medium containing 0.8% sucrose (21 °C, 16 h light). Seven-day-old seedlings were treated with 100 mM or 150 mM NaCl by transferring the seedlings to plates containing 1/2 MS medium and NaCl, and the plants were incubated for either 0 h, 0.5 h, 1 h, or 3 h before RNA extraction.

### RNA extraction and real-time PCR analysis

Seedlings (10–100 mg) were ground into a powder with liquid nitrogen, and 1 ml RezolTM C&T (Omics Bio, Taipei, Taiwan) with 200 μl chloroform was added. The samples were centrifuged at 12,000×*g* (Sigma 1–15 K, USA) for 15 min and moved to a new 1.5 ml tube. Five hundred microliters of isopropanol were added, and the samples were centrifuged at 12,000×*g* (Sigma 1–15 K, USA) for 10 min. The pellets were washed with 75% EtOH and resolved using DEPC-H_2_O. The contaminating DNA was removed using TURBO DNA-free™ DNase according to the manufacturer’s instructions (Ambion, California, USA). Isolated RNA was used for cDNA synthesis using the iScript™ cDNA Synthesis Kit (BIO-RAD, California, USA). Real-time PCR was performed using CFX and CFX manager software (BIO-RAD, California, USA). SsoFast™ EvaGreen Supermix (BIO-RAD, California, USA) was used for amplifications. Arabidopsis *ACTIN2* was used as a quantitative control.

### Isolation of Arabidopsis leaf protoplasts

Arabidopsis protoplast extraction was carried out as previously described and modified (Yoo et al. 2007). Arabidopsis plants were grown in the soil in an environmentally-controlled chamber with a relatively short photoperiod (8 h light at 22 °C/16 h dark at 22 °C). Well-expanded leaves were chosen from 3-week-old plants. One mm leaf strips were cut from the middle part of a leaf using a fresh sharp razor blade. Leaf strips were gently transferred into the prepared enzyme solution (1% cellulose R10, 0.25% macerozyme R10, 0.4 M mannitol, 20 mM KCl, 20 mM MES pH 5.7, 10 mM CaCl_2_, 5 mM β-mercaptoethanol, and 0.1% BSA) by dipping both sides of the strips. The tissues were then placed under a vacuum for 30 min. The digestion reaction continued for at least 3 h at room temperature with shaking. The enzyme solution should turn green after a gentle swirling motion, which indicates the release of protoplasts. A clean filter paper was washed with a W5 solution (154 mM NaCl, 125 mM CaCl_2_, 5 mM KCl, 2 mM MES pH 5.7, and 5 mM Glucose) before protoplast filtration. The enzyme solution containing protoplasts were filtered after wetting the filter paper. The flow-through was centrifuged at 100*g* (2420, KUBOTA, Japan) to pellet the protoplasts in a 15 ml round-bottomed tube for 3 min. The supernatants were removed, and protoplast pellets were washed by gentle swirling with the W5 solution. Protoplasts were resuspended in the W5 solution after counting cells under the microscope using a hemocytometer (BX40, OLYMPUS, USA). The protoplasts were kept on ice for 30 min and then resuspended in an MMG solution (4 mM MES pH 5.7, 0.4 M mannitol, 15 mM MgCl_2_) to a concentration of 2.5 × 10^5^ protoplasts/ml.

### Plasmid construction, and the transformation of plasmids for bimolecular fluorescence complementation (BiFC) analysis

BiFC analyses were carried out by a modified method, as previously described (Yoo et al. 2007; Liu et al. 2012). The fluorescence signal of the yellow fluorescence protein (YFP) was measured for protein–protein interactions. The open reading frame of *AtABF3* was amplified from cDNA. The amplified open reading frame was inserted into the pSAT4-YN or pSAT5-YC vector (from Dr. Kai-Wun Yeh’s lab), driven by the 35S promoter and fused to the YFP-N or YFP-C in frame. The YN and YC fragments of YFP were fused to the C-terminus of the full-length *ABF3* cDNA, and *14*-*3*-*3ω*, respectively. The paired plasmids were transfected into *Arabidopsis* protoplasts (ABF3^N^/14-3-3ω^C^, ABF3^C^/14-3-3ω^N^). ACS7 was used as a positive control for the 14-3-3 interaction  (Huang et al. 2013). Moreover, the transformation of Empty^N^/14-3-3ω^C^, ACS7^C^, Di19-2^C^, ABF3^C^, and Empty^C^/14-3-3ω^N^, ACS7 ^N^, Di19-2^N^, ABF3^N^ were used as negative controls (Additional file 2). Ten microgram plasmids (YFP-N and YFP-C) and 100 μl protoplasts were added into a 15 ml round-bottomed tube and gently mixed. One hundred and ten microliters of a polyethylene glycol solution were added and incubated at room temperature for 10 min. The polyethylene glycol solution containing protoplasts was diluted with 1 ml of the W5 solution and gently mixed. Protoplasts were centrifuged at 100×*g* (KUBOTA 2420, Japan) for 3 min to pellet the protoplasts. The supernatants were removed, and the protoplasts were washed twice with the W5 solution. The protoplasts were resuspended with 100 μl of the W5 solution in an Eppendorf tube at room temperature. After 12–16 h, YFP fluorescence was detected using a confocal microscope (TCS SP5, Leica).

### The transactivation assay using Arabidopsis leaf protoplasts

For the reporter gene construct, the 5× GAL4, 4X GCC, and TATA box in the 5× GAL4-4X GCC-TATA-LUC-Nos M13Fprimer vector was replaced by the *ABI5* promoter and fused to the firefly *Luc* gene. For effector plasmids, the *AtERF* gene in the pUC vector was replaced by the coding regions of *14*-*3*-*3ω*, *ABF3* wild type, mutated *ABF3* (T451A), *ABF3* (T128A), *ABF3* (S126A), and *ABF3* (S134A) that were constructed into the pRTL2 vector using the Gateway LR Clonase™ II Enzyme Mix (Invitrogen, California, USA). The PRL plasmid containing the *Renilla Luc* gene driven by the CaMV35S promoter was used as an internal control for the transactivation assay. Arabidopsis protoplasts were isolated and transfected by a modified polyethylene glycol method, as previously described (Abel and Theologis 1994). Ten micrograms of a reporter plasmid and 5 μg of an effector plasmid were co-transfected into protoplasts with 10 μg of the internal control plasmid, PRL. The transfected cells were incubated at 22 °C for 20 h under light. Protoplasts were harvested by centrifugation at 500×*g* for 1 min (Z223 M-2, HERMLE, Germany). Cells were assayed for luciferase activity using the Dual-Glo™ Luciferase Assay System (Promega) following the manufacturer’s instructions.

### The in vitro kinase assay

Purification of calcium-dependent protein kinasea (CDPKs) (GST-CDPK3-6H, GST-CDPK16-6H) and substrates (wild type and mutant variants of GST-GFP-Strep-tagged fusion peptides as shown in Table 1) was carried out as previously described (Curran et al. 2011). The in vitro kinase assay was carried out as previously described (Curran et al. 2011). 50 μM of an ATP solution (spiked with 2.5 μCi [γ-^32^P] ATP) was added into the reaction mixture, which consisted of a 0.25 μg purified recombinant calcium-dependent protein kinase, 5 μg fusion protein substrate, and standard kinase reaction buffer to start the kinase reaction. The reaction tubes were incubated at RT for 10 min, and the reactions were stopped by adding 5 μl of a 5× sodium dodecyl sulfate (SDS) sample buffer. All samples were loaded into the 10% SDS-PAGE loading well for electrophoresis, and the gel was air-dried with acetate sheets. The γ-^32^P-labeled signals were normalized with the amount of protein as determined from Coomassie Brilliant Blue stained gels after running SDS-PAGE. The γ-^32^P-labeled signals were detected with an image analyzer (Typhoon 9400).

**Table 1:**
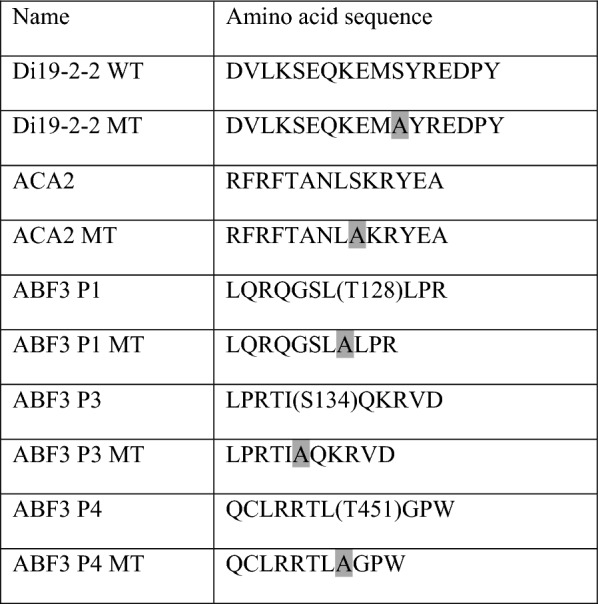
The fusion peptides used in the kinase assay

Gray bars represent point mutations of amino acid residues by site-directed mutagenesis

### Chromatin immunoprecipitation (ChIP) assays

Two-week-old seedlings grown on vertically oriented plates with MS medium were collected (~ 2 g) for the ChIP assays (Gendrel et al. 2005). After fixation with formaldehyde, the chromatin was sheared to an average length of 500 bp by sonication and then immunoprecipitated with Protein G Mag Sepharose Xtra magnetic beads (GE) and an anti-YFP antibody (catalog: 66002-1-lg, proteintech). After the cross-linking was reversed, the number of precipitated DNA fragments and amount of input DNA was detected by quantitative real-time PCR using the specific primers. The percentage of input DNA was calculated by determining 2^−ΔCt^(= 2^−[Ct(ChIP)−Ct(Input)]^). *ACTIN2* and *UBQ10* were used as the negative control.

## Results

### The survival rate of the *abf3* mutant is decreased under 150 mM NaCl salt stress condition

**Fig. 1:**
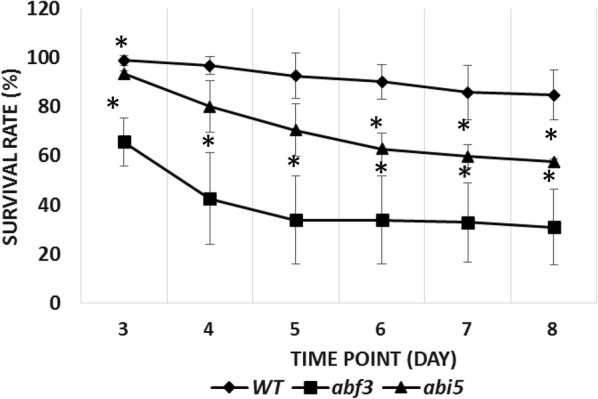
The survival rates of *abf3* and *abi5* mutants under salt stress conditions. Survival rates of the Arabidopsis *abf3* and *abi5* mutants with 150 mM NaCl treatments. Plants grown on half-strength MS plates for 3 days were transferred to a medium containing 150 mM NaCl for 8 days, and then images were taken. N = 3, n ≥ 33. Significant differences were defined at *P < 0.05 and **P < 0.001 by the Student’s *T* test

To compare the salt tolerance between the Col-0 and T-DNA insertion mutant, *abf3*, 3-day-old seedlings were subjected to 150 mM NaCl for 8 days to observe survival rates. Lack of bleaching was used to score the survival rate. After transferring seedlings to the half-strength MS medium containing 150 mM NaCl for 3 days, the Col-0 plants showed better growth and a higher survival rate than the *abf3* mutant lines. About 90% of the NaCl-treated Col-0 plants survived while only around 40% of the *abf3* mutant line seedlings and 60% of the *abi5* mutant line seedlings survived after being treated with salt for 8 days (Fig. 1). These results indicated that *ABF3* and *ABI5* loss-of-function mutations decreased a plant’s tolerance to salt stress.

### Altered *ABI5* expression in the *abf3* mutants under salt stress condition

**Fig. 2:**
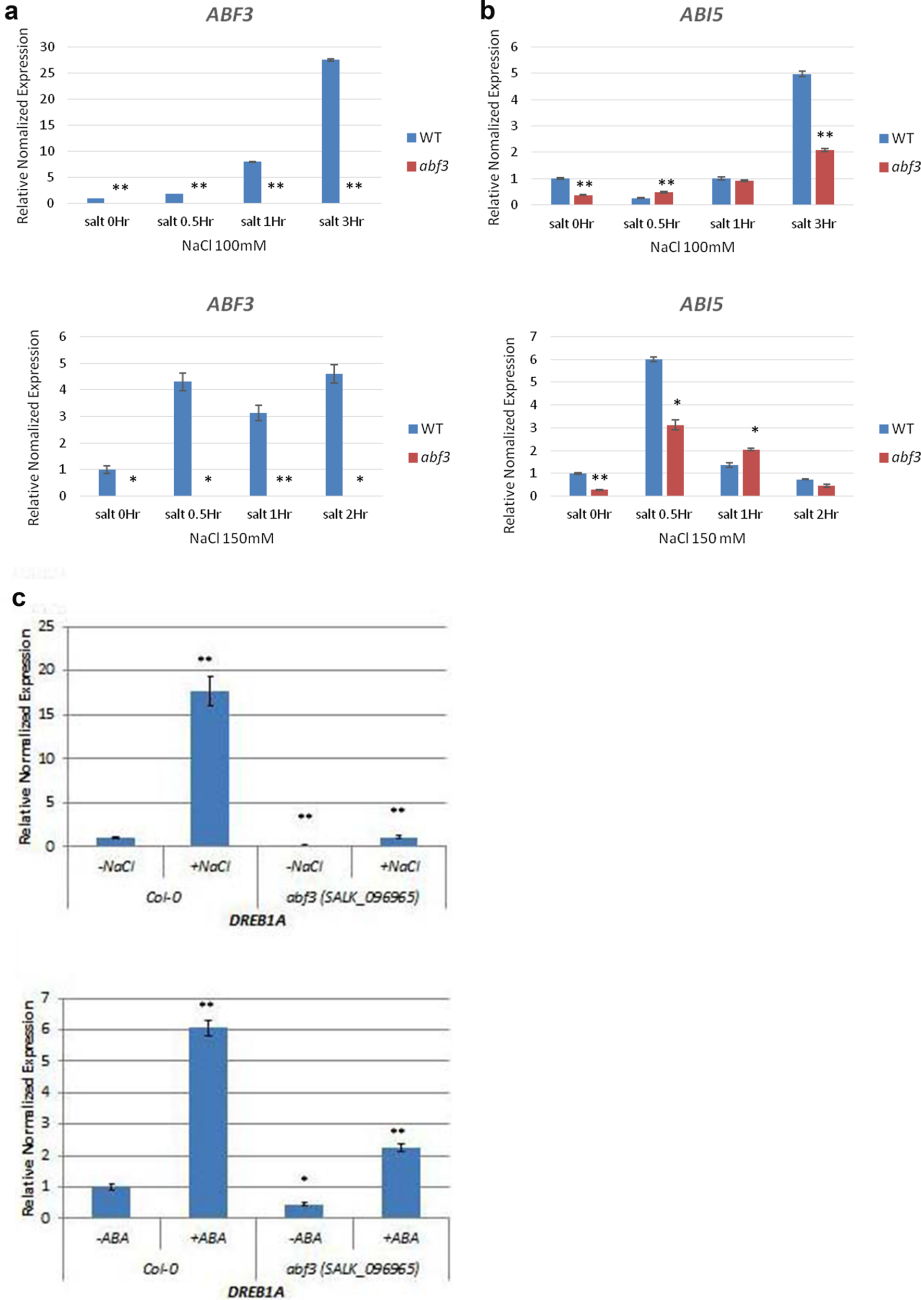
*ABI5* gene expression in *abf3* mutants under salt stress conditions. Real-time PCR was used to investigate if *ABF3* and *ABI5* could be regulated in the *abf3* mutant background (SALK_096965) under salt stress conditions. **a** At 0 h, 0.5 h, 1 h, and 3 h, *ABF3* was not upregulated in the mutant *abf3* background with 100 mM of salt. **b** At 0.5 h, 1 h, and 2 h, *ABI5* was upregulated in the mutant *abf3* background with 150 mM of salt. **c** At 3 h, *DREB1A* stress-related gene expression was up-regulated in the *abf3* mutant background with 100 mM of salt. Data are presented as the mean ± standard deviation (SD). Significant differences between the WT plants and *abf3* mutants are indicated by *P < 0.05 and **P < 0.001 using the Student*’*s T-test

To investigate whether *ABF3* regulates *ABI5* under normal and salt stress conditions, quantitative RT-PCR (qRT-PCR) was used to analyze *ABI5* gene expression in the *abf3* mutant. The stress marker gene, *DREB1A,* was up-regulated in 100 mM and 150 mM NaCl salt stress conditions. Under 100 mM or 150 mM NaCl salt stress condition, *ABI5* was up-regulated in the *abf3* mutant. However, the expression levels were still lower than wild type under salt stress conditions (Fig. 2a, b). These results indicated that *ABI5* expression is regulated by salt in Arabidopsis, and suggests that the regulation of *ABI5* by *ABF3* involves in the salt stress response.

### ABF3 phosphorylation is catalyzed by CDPK3 and CDPK16 in vitro

**Fig. 3:**
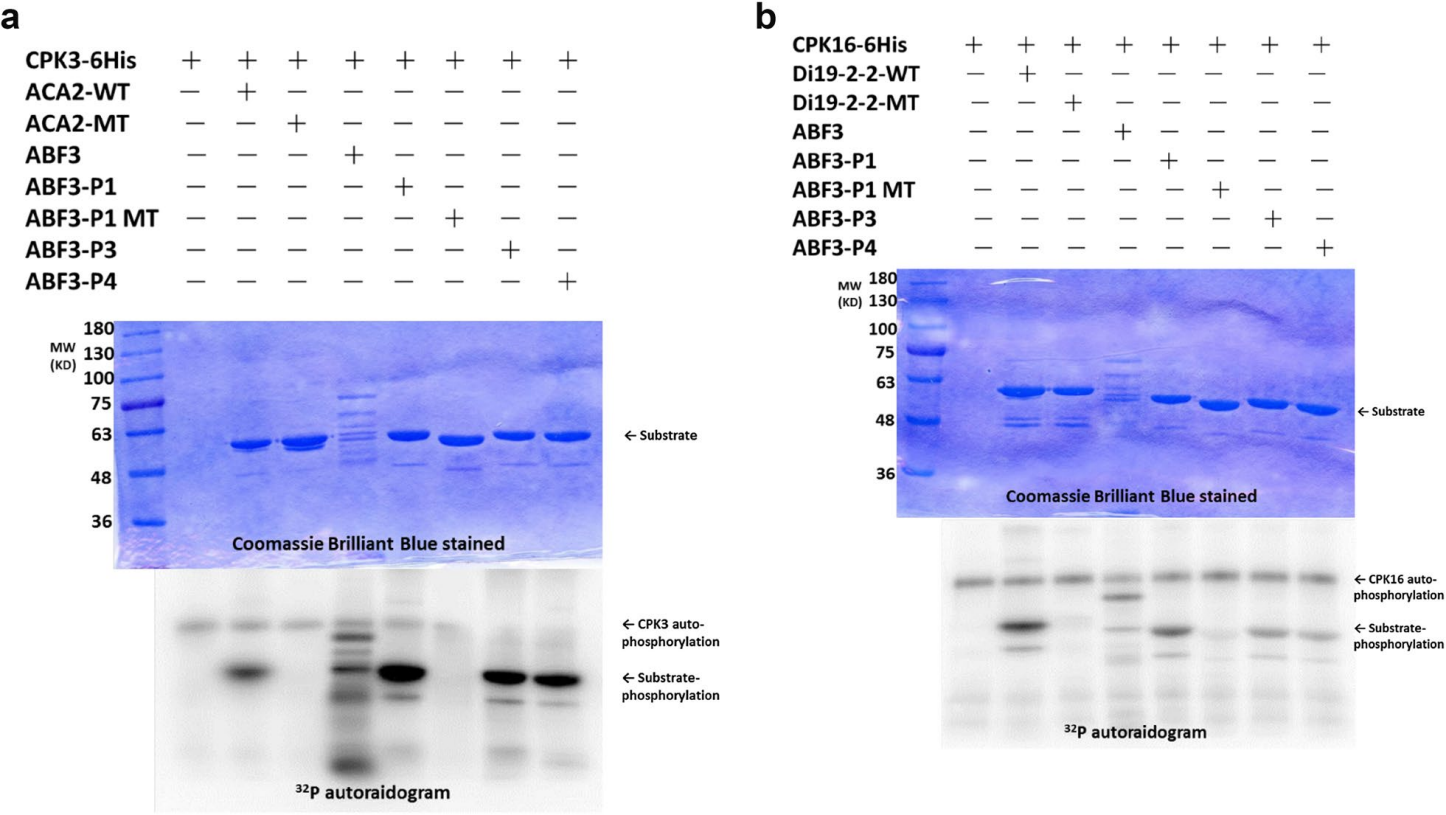
AtABF3 is phosphorylated by AtCDPKs at its phosphorylation site, in vitro. Recombinant fusion peptides [ABF3 P1 (LQRQGpSLpTLPR), ABF3 P1 MT (LQRQGpSLALPR), ABF3 P3 (LPRTIpSQKRVD), and ABF3 P4 (QCLRRTLpTGPW)] were used as substrates, and two recombinant kinases (AtCDPK16-6His, AtCDPK3-6His) were used to perform the kinase assay. The molecular weight of the full-length recombinant GST-ABF3 protein is about 76kD. The molecular weights of several different fusion proteins are about 55 kD. The fusion peptide phosphorylation signal is marked with an arrowhead, and the kinase auto-phosphorylation signal is about 100 kD. The results showed that that ABF3 could be phosphorylated by **a** AtCDPK3-6His and **b** AtCDPK16-6His, in vitro. The ACA2 wild-type (WT) is a fusion peptide in which the GST protein is fused with a peptide from ACA2, RFRFTANLpSKRYEA, which could be recognized by AtCDPK3-6His. The ACA2 mutant (MT) is similar to ACA2 WT, but a Serine was mutated to Alanine. Di19-2-2 is a fusion peptide with a GST protein that is fused with a peptide (DVLKSEQKEMpSYREDPY), and this peptide is recognized by AtCDPK16-6His

Since CPK3 was reported to be involved in the salt stress response (Mehlmer et al. 2010) and CPK16 might have substrate specificity (Curran et al. 2011), recombinant CPK3 (GST-CDPK3-6H) and CPK16 (GST-CDPK16-6H) were used for the kinase assay. According to our predicted conserved Ser/Thr residues of ABF3, fusion peptides containing these candidate sites were designed for the kinase assay in vitro (Table 1). It was found that the full-length ABF3 and fusion peptide containing the fragment of ABF3 with P1 (LQRQGSLTLPR), P3 (LPRTISQKRVD), and P4 (QCLRRTLTGPW) were labeled using ^32^P in the autoradiograms (Fig. 3). The results indicated that the ABF3 fusion peptide could be phosphorylated by recombinant GST-CDPK3-6His (Fig. 3a) and GST-CDPK16-6His (Fig. 3b) in vitro. Because ABF3 P1 contained one serine and one threonine (Ser-126 and Thr-128) as potential phosphorylation sites, it was not known which amino acid is the actual CDPK phosphorylation site. Therefore, we created point mutations (threonine to alanine in ABF3 P1-MT) (Table 1). The results indicated that P1, P3, and P4 could be phosphorylated by CDPKs in vitro. To confirm that the labeling was specifically by CDPK, a mutated (kinase dead) recombinant GST-CDPK16-6His (Curran et al. 2011) was used in the kinase assay. The phosphorylation of recombinant GST-ABF3 was abolished (Additional file 3). Taken together, our results indicated that full-length ABF3 could be phosphorylated by CDPKs, and Thr128, Ser134, and Thr451 of ABF3 are CDPK in vitro phosphorylation sites.

### Nuclear protein–protein interactions between ABF3 and 14-3-3ω were detected using BiFC analysis

**Fig. 4:**
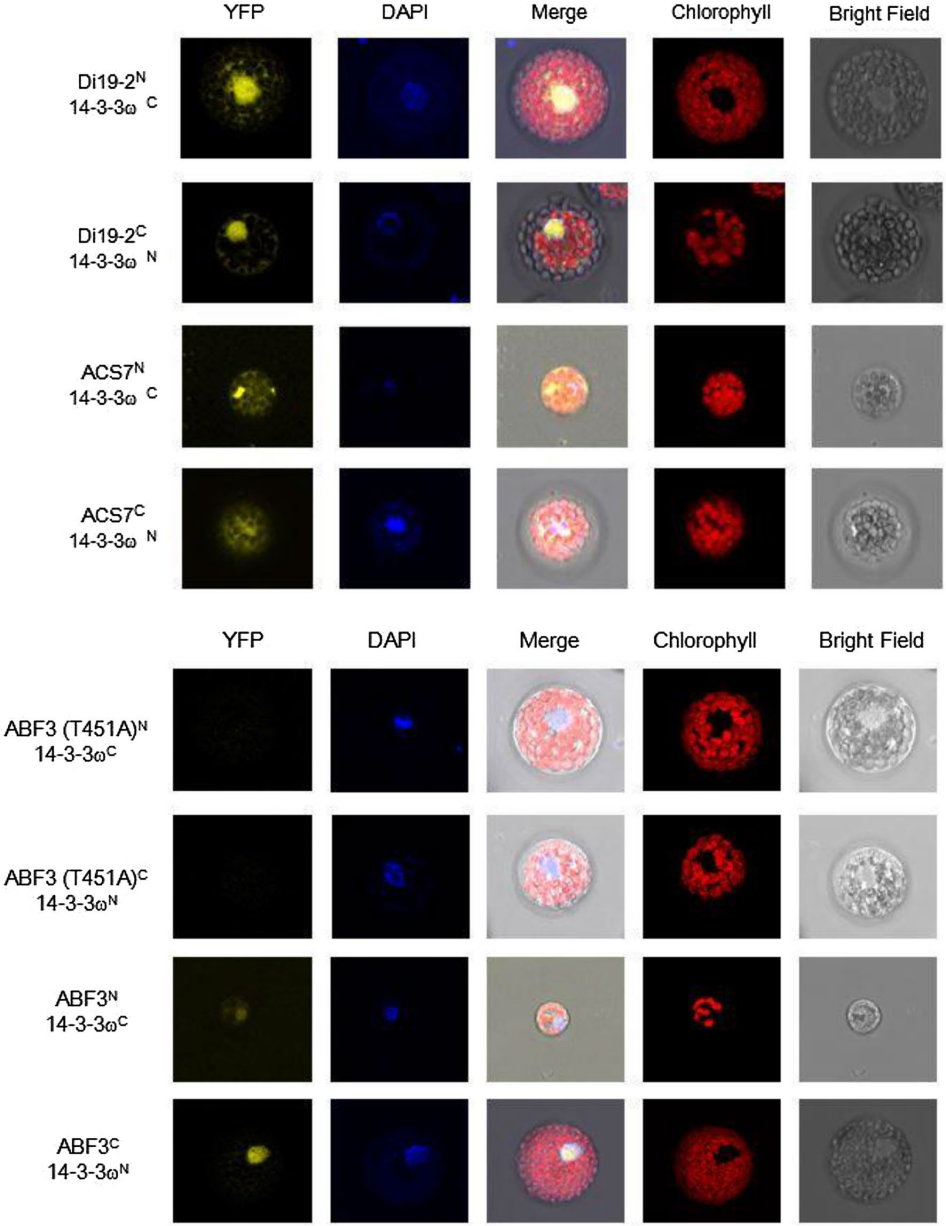
ABF3 and 14-3-3ω protein–protein interactions are revealed using bimolecular fluorescence complementation (BiFC). The 14-3-3ω, Di19-2, ACS7, ABF3 proteins were paired with empty vectors used as negative controls. The interaction between YFP^N^-Di19-2 and YFP^C^-14-3-3ω/YFP^C^-Di19-2 and YFP^N^-14-3-3ω/YFP^N^-ACS7 and YFP^C^-14-3-3ω/YFP^C^-ACS7 and YFP^N^-14-3-3ω was localized to the nucleus and cytosol as shown in the confocal microscopy images. DAPI was used to stain the nucleus. ACS7 interacts with 14-3-3, and therefore, was introduced as a positive control (Huang et al. 2013). BiFC experiments showed an interaction between ABF3 and 14-3-3ω in *Arabidopsis* protoplasts. Point mutations of ABF3 (T451A) abolished its interaction with 14-3-3ω. DAPI was used to stain the nucleus

The 14-3-3 protein is a scaffold protein that associates with many different cellular proteins of which most are phosphorylated (Chang et al. 2009; Paul et al. 2009; Jaspert et al. 2011). A previous study showed that ABF3 could interact with 14-3-3 in vitro using a pull-down assay (Sirichandra et al. 2010). In the present study, BiFC was introduced to test the interaction between ABF3 and 14-3-3ω. The yellow fluorescence signal of ABF3^N^/14-3-3ω^C^ and ABF3^C^/14-3-3ω^N^ were observed in the nucleus, indicating that 14-3-3ω and ABF3 had a physical interaction (Fig. 4). Based on the transient expression results, ABF3 was found to physically interact with 14-3-3ω in the nucleus.

To identify the ABF3 binding site, the mutation of a predicted 14-3-3 binding site to Ala (T451A) in ABF3 was tested to see if there was an interaction with 14-3-3. No interaction between ABF3 (T451A) and 14-3-3 was detected (Fig. 4). The transient expression results confirmed that the binding site was the T451 of ABF3, which is consistent with the results of Sirichandra et al. (2010), who showed that the T451 of ABF3 is the 14-3-3 binding site.

**Fig. 5:**
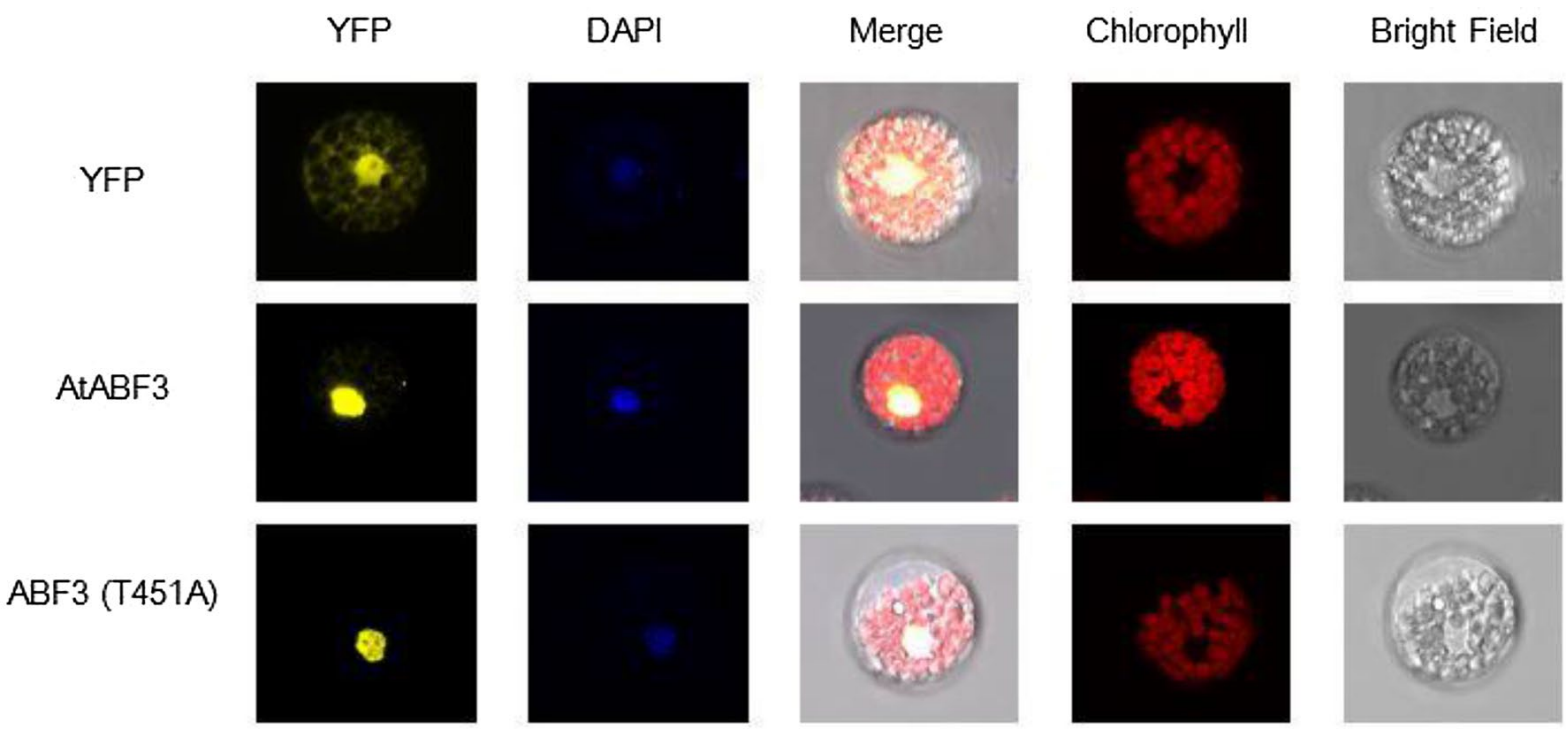
Subcellular localization of mutated ABF3 (T451A). Subcellular localization of mutated ABF3 (T451A) in *Arabidopsis* protoplasts was measured using laser confocal microscopy. The ABF3 T451 was mutated to Ala. DAPI was used to stain the nucleus

The 14-3-3 binding site was hypothesized to interact with ABF3 to affect protein localization or regulate transcriptional activity. Mutated ABF3 (T451A), which lacked the conserved 14-3-3 binding sequence, was YFP-fused and transfected into *Arabidopsis* protoplasts. The YFP fluorescent signal was still located in the nucleus for both the ABF3 and mutated ABF3 (T451A) (Fig. 5). The subcellular localization of ABF3 and mutated ABF3 (T451A) proteins were not different, indicating that the mutation of 14-3-3 binding sequence does not likely change the subcellular location of the ABF3 protein.

### *ABI5* activation by either ABF3 alone or ABF3 co-expressed with 14-3-3ω in a transactivation assay

**Fig. 6:**
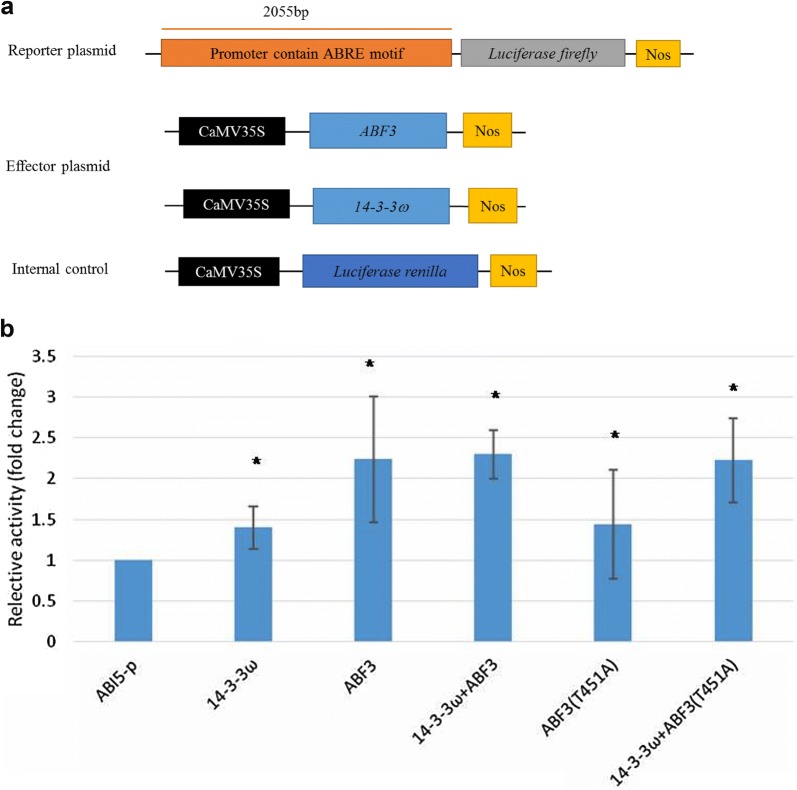
*ABI5* expression is activated in the presence of ABF3 and 14-3-3 as measured by the transactivation assay. Schematic diagrams of the reporter, effector, and internal control plasmids of the mesophyll protoplasts used in transient transactivation assays. **a** The reporter plasmid contained the *ABI5* promoter (2055 bp). The effector plasmids contained the *ABF3 and 14*-*3*-*3ω* gene, which were driven by the *CaMV35S* promoter. The PRL vector contained a *CaMV35S* promoter driving the Renilla luciferase gene, *Luc,* as an internal control. **b** The results showed that *ABI5* is regulated by ABF3 and 14-3-3*ω*. Data are presented as the mean ± standard deviation (SD). N = 3, three biological repeats with five technical replicates for each biological repeat. Relative activity is calculated as the LUC firefly/LUC Renilla but is normalized to the reporter only. The fold change of the relative activity significantly differed from the reporter only as indicated by *P < 0.05 using the Student’s T-test

The *ABI5* gene promoter (started from the translation start site) was analyzed by PLANTCARE (http://bioinformatics.psb.ugent.be/webtools/plantcare/html/) or AGRIS (http://arabidopsis.med.ohio-state.edu). Four ABREs were predicted from − 958 to − 2295 of the *ABI5* promoter region. To investigate the relationship between *ABI5* and ABF3, a transactivation assay was performed. The reporter plasmid harboring the *ABI5* promoter (2055 bp) sequence fused with a firefly luciferase reporter gene was constructed. Effector plasmids were constructed with the *ABF3* and *14*-*3*-*3ω* genes each driven by the CaMV35S promoter. The PRL plasmid containing the Renilla luciferase gene driven by the CaMV35S promoter was used as an internal control. In the transactivation assay, the reporter, effector, and internal control plasmid were co-transfected into Arabidopsis protoplasts, and luciferase signal detection was used to determine transcriptional regulation. Transient luciferase expression indicated that *ABI5* was activated when ABF3 alone or ABF3 combined with 14-3-3ω were expressed (Fig. 6).

To determine if *ABI5* activation was induced by 14-3-3ω, mutated ABF3 (T451A) was used as an effector and luciferase expression was observed. Compared with the wild type ABF3, mutated ABF3 (T451A) combined with 14-3-3ω did not activate *ABI5* (Fig. 6). Therefore, T451 phosphorylation might not be required for ABF3-promoted *ABI5*::*LUC* activity under normal conditions. T451 phosphorylation of ABF3 by the ABA-activated kinase, OST1, was previously shown to be required for ABF3 stability in Arabidopsis (Sirichandra et al. 2010). When the T451 site on of ABF3 was point-mutated into an Ala, ABF3 stability was affected (Sirichandra et al. 2010). Since our results showed that T451 is not important for ABF3 transcriptional activation, T451 phosphorylation and 14-3-3 binding could exclusively function in the regulation of ABF3 protein stability (Sirichandra et al. 2010). Taken together, these results indicate that ABF3 regulates *ABI5* expression under normal conditions.

### A promoter deletion assay identified ABRE *cis*-elements of the *ABI5* promoter in a transactivation assay

**Fig. 7:**
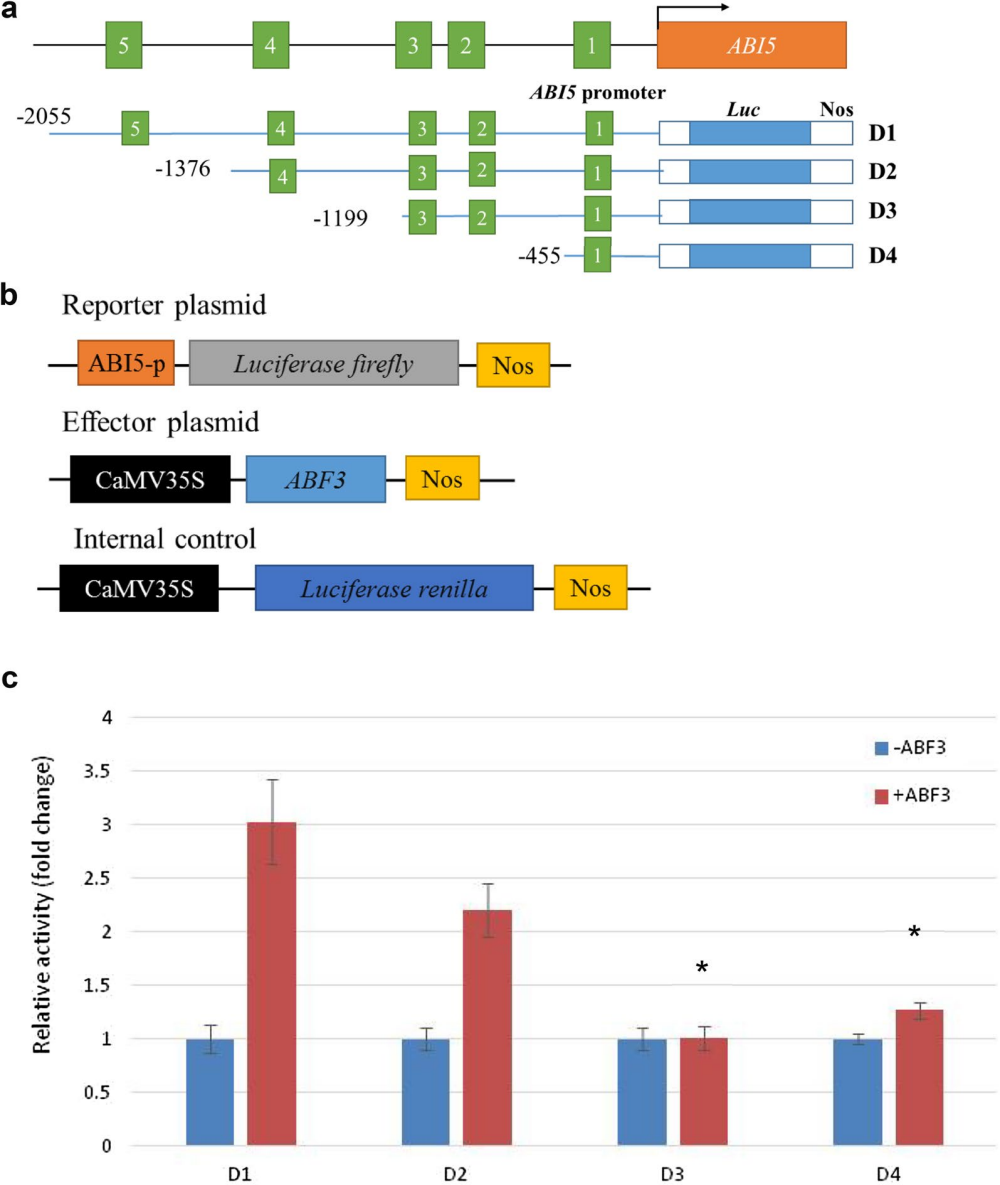
The *ABI5* promoter deletion assay. Schematic diagrams of the reporter, effector, and internal control plasmids used in the transient transactivation assay in *Arabidopsis* leaf protoplasts. **a** The reporter plasmid contained the *ABI5* promoter. For the effector plasmid, the *ABF3* gene was driven by the *CaMV35S* promoter. The PRL vector contained a *CaMV35S* promoter that drove the Renilla luciferase gene, *Luc,* as an internal control. **b** A schematic diagram of constructs of the *ABI5* promoter with 5′ end deletions fused to the *Luc* reporter gene. D1 contains an ABRE *cis*-element (BOX 1 to BOX 5). D2 contains an ABRE *cis*-element (BOX 1 to BOX 4). D3 contains an ABRE *cis*-element (BOX 1 to BOX 3). D4 contains an ABRE *cis*-element (BOX 1). **c** The results indicated that through the deletion of the *ABI5* promoter, transcriptional activity was decreased. ABF3 bound to BOX4 and BOX5 of the ABRE *cis*-element to regulate *ABI5* transcription. Data are presented as the mean ± standard error (SE). N = 3, three biological repeats with three technical replicates for each biological repeat. The relative activity is calculated as the LUC firefly/LUC Renilla but is normalized to the minus ABF effector. The relative activity fold-change significantly differed from D1 is indicated by *P < 0.05 using the Student’s T-test

Through *ABI5* promoter sequence (− 1 to − 2055) analyses, five predicted ABRE *cis*-elements (CACGTG) (Additional file 4) were found. To study which *cis*-element can be regulated by ABF3, constructs of the *ABI5* promoter with 5′ end deletions were generated. Figure 7 shows D1 with a full-length promoter sequence (− 1 to − 2055) containing BOX 1 to BOX 5. BOX 5 was deleted in D2, and the sequence of the promoter was − 1 to − 1376. BOX 5 and BOX 4 were deleted in D3, and the sequence of the promoter was − 1 to − 1199. D4 contained only BOX 1, and the sequence of the promoter was − 1 to − 455. Since BOX 2 and BOX 3 were quite close to one another, both were deleted simultaneously. Reporter plasmids harboring different lengths of the *ABI5* promoter sequence fused with a firefly luciferase reporter gene were constructed. Effector plasmids were constructed with *ABF3* driven by the CaMV35S promoter. The PRL plasmid containing the Renilla luciferase gene driven by the CaMV35S promoter was used as an internal control. The results indicated that through the deletion of boxes in the *ABI5* promoter region, transcriptional activity was decreased (Fig. 7). In particular, BOX4 and BOX5 appeared to be the ABRE *cis*-element that can be bound by ABF3 to regulate *ABI5* expression under normal condition.

### ABF3 binds to the *ABI5* promoter in vivo under salt stress condition

**Fig. 8:**
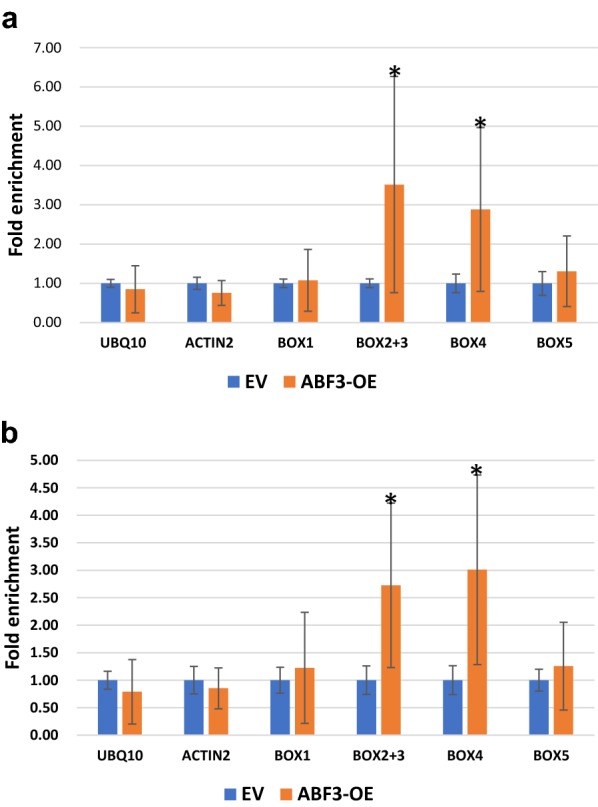
ABF3 binds to the *ABI5* promoter in vivo. The ChIP-qPCR analysis of the ABI5 promoter regions using the immunoprecipitation of the yellow fluorescent protein (YFP) in ABF3-overexpression lines using pEarleyGate 101 as the vector system. *ABF3*-*YFP* in Col is a transgenic line with YFP-tagged ABF3 expression in a Col background. Plants were grown on half-strength MS plates for 14 days followed with no treatment (**a**) or with 150 mM NaCl treatment (**b**) by 3 h. The enrichment of BOX 1 to BOX 5 was revealed by real-time PCR. *UBQ10* and *ACTIN2* were used as a negative control. Data are presented as the mean ± standard deviation (SD). N = 2, there were three technical replicates for each biological repeat (a total of n = 6). Significant differences between the vector only and *ABF3* overexpression vectors are indicated by *P < 0.05 using the Student*’*s *t*-test

Since *abf3* and *abi5* mutant lines are both salt intolerant (Fig. 1), we hypothesized that ABF3 regulates *ABI5* in response to salt stress in Arabidopsis. To study whether *ABI5* is a direct target of ABF3 in vivo, the ChIP assay was performed using transgenic plants expressing yellow fluorescent protein (YFP)-tagged ABF3 driven by the 35S promoter (*35S:ABF3*-*YFP*). To study which ABRE *cis*-elements can be bound by ABF3, the *ABI5* promoter was divided into BOX 1 to BOX 5, as shown in Fig. 7. The result, shown in Fig. 8, demonstrated that ABF3 bound to the promoter of BOX2, BOX3, and BOX 4 with no treatment. In addition, ABF3 bound to BOX 2, BOX 3, and BOX 4 under 150 mM NaCl salt stress condition, which indicated that *ABI5* is a direct target gene of ABF3.

## Discussion

### The 14-3-3 binding site, T451, is phosphorylated by CDPKs in vitro

ABFs have been reported to be phosphorylated by many kinases. In rice, TRAB1 (a rice ABF) has been shown to be phosphorylated in an ABA-dependent manner (Kobayashi et al. 2005). In potatoes (*Solanum tuberosum*), StABF1 is phosphorylated in response to ABA and salt stress. Specifically, a potato CDPK isoform (StCDPK2) phosphorylated StABF1 *in* *vitro* (Muniz Garcia et al. 2012). In Arabidopsis, AtCPK32 phosphorylated ABF4 in vitro. Serine-110 of ABF4 was phosphorylated by AtCPK32 (Choi et al. 2005). Moreover, a previous study showed that ABF3 could be phosphorylated by SnRK2E/SnRK2.6 in Arabidopsis (Sirichandra et al. 2010). In Fig. 3, GST-ABF3 was found to be phosphorylated by recombinant CDPK3 and CDPK16 in vitro. In addition, the results from our kinase assay showed that Thr-128, Ser-134, and T451 of AtABF3 are CDPK phosphorylation sites. Our results are consistent with these reports with one exception being that Thr-128 is a previously uncharacterized site. According to the conserved kinase domain sequence, CDPK belongs to the CDPK-SnRK superfamily. The CDPK-SnRK superfamily consists of seven serine-threonine protein kinase types, namely calcium-dependent protein kinase (CDPKs), CDPK-related kinases (CRKs), phosphoenolpyruvate carboxylase kinases (PPCKs), PEP carboxylase kinase-related kinases (PEPRKs), calmodulin-dependent protein kinases (CaMKs), calcium and calmodulin-dependent protein kinases (CcaMKs), and SNF-related serine/threonine-protein kinases (SnRKs) (Hrabak et al. 2003). To date, three consensus CDPK phosphorylation motifs have been identified (Jaspert et al. 2011). The phosphorylation site of the fusion peptide, P4, is consistent with the CDPK phosphorylation (φ_-5_-X_-4_-Basic_-3_-X_-2_-X_-1_-S,) and SnRK2 phosphorylation motifs (LXRXX(S/T) (Sirichandra et al. 2010). However, whether AtABF3 is a CDPK substrate, and Thr-128, Ser-134, and Thr-451 are the in vivo phosphorylation sites requires further studies.

### The *ABI5* promoter is regulated by ABF3 binding

ABA signal transduction, perceived from environmental cues to physiologic responses, involves many components, including ABA receptors, protein kinases, phosphatases, transcription factors, and ABA-induced genes containing conserved G–box like *cis*-acting elements (ABREs) in their promoter regions (Hernandez Sebastia et al. 2004). Most ABA-regulated genes contain conserved ABA-responsive elements as the determinant *cis*-elements in their promoters. In general, a single ABRE copy is not able to induce ABA-mediated transcription. Successful ABA-induced gene expression requires either additional copies of ABREs or coupling elements (Shen 1996). Recently, however, a frequency distribution approach has shown that ABRE–ABRE pairs are major *cis*-elements in Arabidopsis and rice (Gomez-Porras et al. 2007). Multiple ABREs or a combination of an ABRE with a so-called coupling element (CE) can establish a minimal ABA-responsive complex (ABRC), and thereby, confer ABA responsiveness to a minimal promoter (Gomez-Porras et al. 2007).

In the ChIP assay (Fig. 8), ABF3 bound to the promoter of BOX 2 + BOX 3 and BOX 4, indicating that *ABI5* is a direct target gene of ABF3. A previous study indicated that *ABI5* and *ABF3* in some seedlings have redundant ABA and stress responses, but the relative importance of these genes varies among responses (Finkelstein et al. 2005). For example, *ABI5* is a much more critical determinant of germination sensitivity to ABA or other stresses, consistent with its much stronger expression in mature seeds. Alternatively, *ABF3* is more important for the ABA sensitivity of seedling root growth (Finkelstein et al. 2005). By contrast, our data showed that ABF3 could directly regulate *ABI5* expression, which has not been previously reported. In addition, ABF3 could bind to the *ABI5* promoter under normal and 150 mM NaCl salt stress conditions (Fig. 8). Under salt stress conditions, the fold enrichment is higher than normal conditions. Our results supported that ABRE–ABRE pairs could be involved in the regulation of *ABI5* gene expression by ABF3 in response to salt stress.

Based on our results, it is possible that AtCDPK3 and AtCDPK16 are activated under salt stress condition. The activated CDPKs may phosphorylate AtABF3 at T451 followed by 14-3-3 binding. The 14-3-3 binding stabilizes ABF3 and ABF3 binds to the promoter region of *AtABI5*, which in turn activates the *ABI5* gene in response to salt stress. In summary, our results showed that ABF3 is an in vitro CDPK substrate. In addition, ABF3 could bind to *ABI5* ABRE elements to activate *ABI5* gene expression in Arabidopsis in response to salt stress.

## Additional files

**Additional file 1.** Isolation of the *abf3* mutant line.

**Additional file 2.** Negative controls for the bimolecular fluorescence complementation analysis.

**Additional file 3.** The in vitro kinase assay of GST-ABF3 phosphorylated by mutated GST-CDPK16-6His.

**Additional file 4.** The predicted ABRE *cis*-elements in the *ABI5* promoter sequence (− 1 to − 2055).

## Data Availability

Agree.
