# Supplementary material for: Regulation of ABI5 expression by ABF3 during salt stress responses in Arabidopsis thaliana
Source: Bot Stud. 2019 Aug 9;60:16. doi: 10.1186/s40529-019-0264-z (PMC6689043; doi:10.1186/s40529-019-0264-z)
Supplement: Supplementary file 1 — Additional file 1. Isolation of the abf3 mutant line. [file 40529_2019_264_MOESM1_ESM.pdf]

Additional files

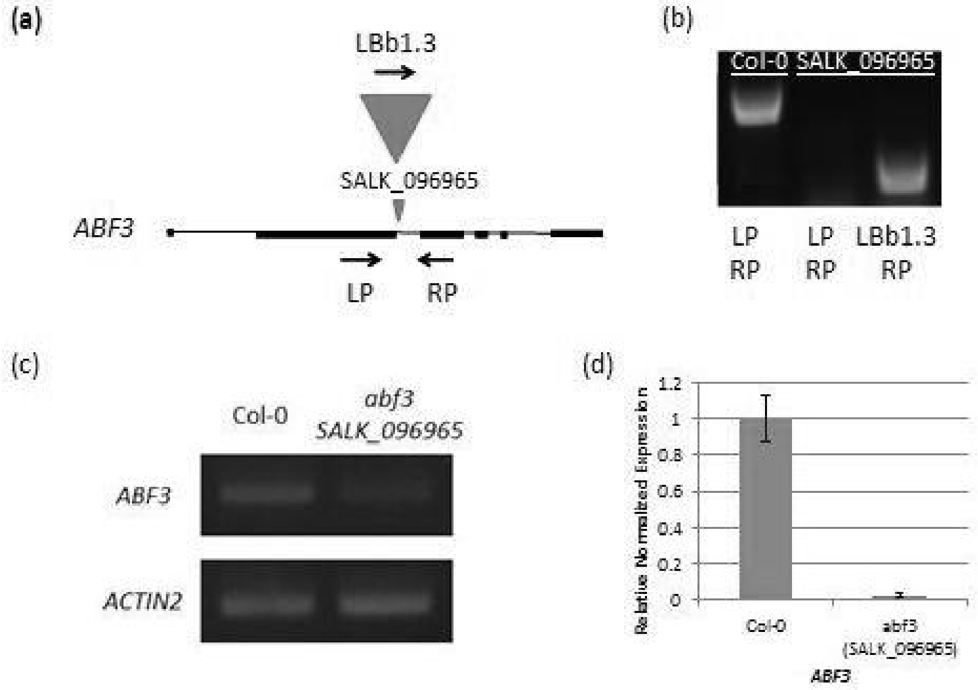

Additional file 1: Isolation of *abf3* mutant line.

(a) Primer design for genotyping, LBb1.3: ATTTTGCCGATTTCGGAAC, LP: ACACTGTTATTAACGGCGGTG, RP: CTTTCTCCAGAACTGCACCTG

(b) Genomic DNA of Col-0 and SALK_096965 was isolated, and specific primer, LP, RP and LBb1.3 were used to confirm the T-DNA insertion line is homozygous or not.

(c) RT-PCR analysis of ABF3 transcript in wild-type and *abf3* mutant. The same amount of cDNA was used for PCR analysis using primer for *ABF3* (30 cycles), *ACTIN2* (30 cycles).

(d) Real-time PCR analysis showed ABF3 gene expression in wild-type and *abf3* mutant.
