# Supplementary material for: Regulation of ABI5 expression by ABF3 during salt stress responses in Arabidopsis thaliana
Source: Bot Stud. 2019 Aug 9;60:16. doi: 10.1186/s40529-019-0264-z (PMC6689043; doi:10.1186/s40529-019-0264-z)
Supplement: Supplementary file 2 — Additional file 2. Negative controls for the bimolecular fluorescence complementation analysis. [file 40529_2019_264_MOESM2_ESM.pdf]

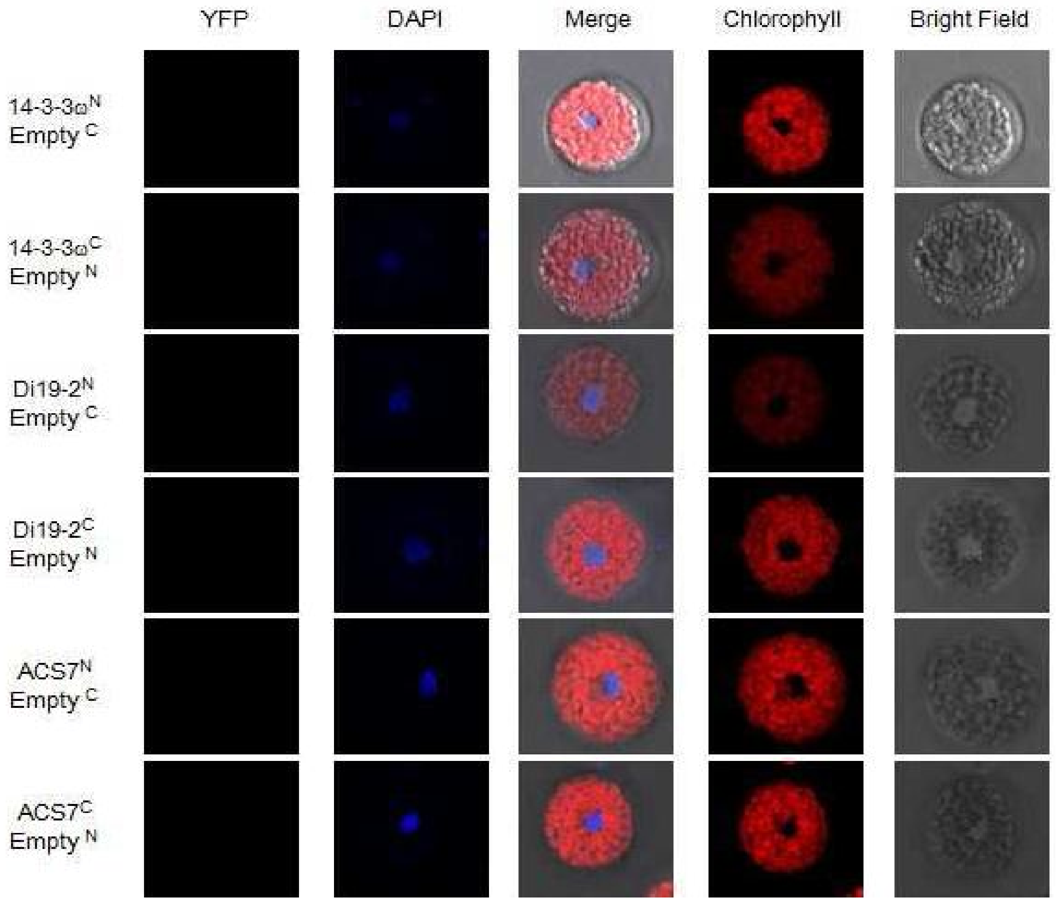

Additional file 2. Negative controls for the bimolecular fluorescence complementation analysis.
