# Supplementary material for: Regulation of ABI5 expression by ABF3 during salt stress responses in Arabidopsis thaliana
Source: Bot Stud. 2019 Aug 9;60:16. doi: 10.1186/s40529-019-0264-z (PMC6689043; doi:10.1186/s40529-019-0264-z)
Supplement: Supplementary file 3 — Additional file 3. The in vitro kinase assay of GST-ABF3 phosphorylated by mutated GST-CDPK16-6His. [file 40529_2019_264_MOESM3_ESM.pdf]

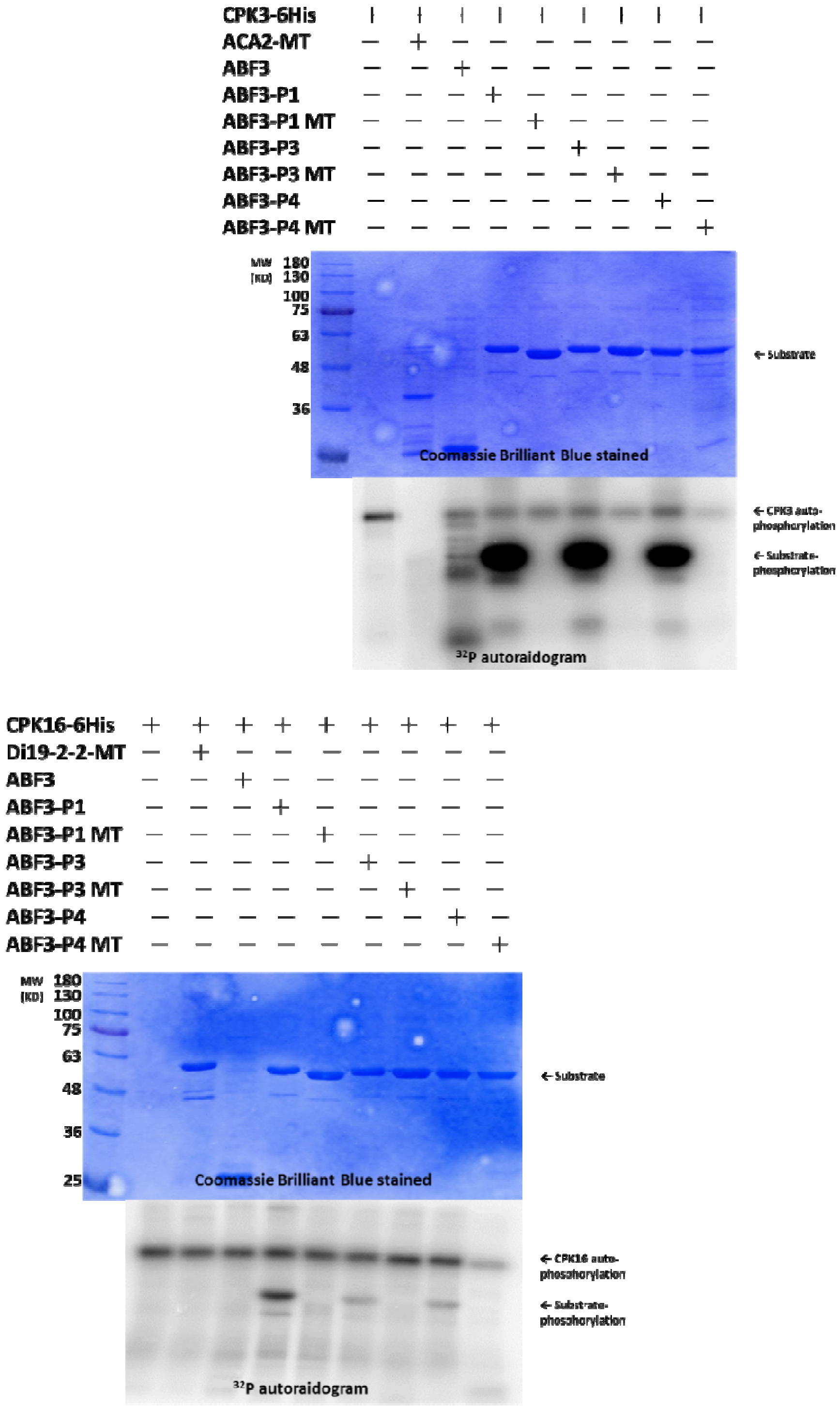

Additional file 3. The *in vitro* kinase assay of GST-ABF3 phosphorylated by mutated GST-CDPK16-6His. Recombinant fusion peptides ABF3 P1 〔(LQRQGpSLpTLPR), ABF3 P1 MT (L QRQGpSLALPR), ABF3 P3 (LPRTIpSQKRVD), ABF3 P3 MT (LPRTIAQKRVD), ABF3 P4 (QCLRRTLpTGPW) and ABF3 P4 MT (QCLRRTLAGPW) 〕 were used as substrates, and two recombinant kinases (AtCDPK16-6His, AtCDPK3-6His) were used to perform the kinase assay *in vitro*.
