# Supplementary material for: Regulation of ABI5 expression by ABF3 during salt stress responses in Arabidopsis thaliana
Source: Bot Stud. 2019 Aug 9;60:16. doi: 10.1186/s40529-019-0264-z (PMC6689043; doi:10.1186/s40529-019-0264-z)
Supplement: Supplementary file 4 — Additional file 4. The predicted ABRE cis-elements in the ABI5 promoter sequence (− 1 to − 2055). [file 40529_2019_264_MOESM4_ESM.pdf]

cis-element	Region	Sequences
W box	-1046~-1051	AGACAAAAGATG**TTGACC**TTCACGCCTCTC
ABRE (BOX2)	-1154~-1161	ACGTGCAGGA**CACGTGTC**GTCCGCAGCCGA
ABRE (BOX3)	-1167~-1172	GCCGAAGTCA**CACGTGTC**GAGCCTGTGAGA
ABRE (BOX4)	-1211~-1218	GCCGAAGTCA**CACGTGTC**GAGCCTGTGAGA
W box	-1462~-1467	TGTTTACAAACT**TTGACT**ATTTCTTTCATA
ABRE (BOX5)	-1689~-1694	CAGTGAATAGTC**CACGTG**CACTCCCAATGG

cis-element of ABREs or W-boxes are in bold face.

Additional file 4. The predicted ABRE *cis*-elements in *ABI5* promoter sequence (-1 to -2055).
